# A novel 4 immune-related genes as diagnostic markers and correlated with immune infiltrates in major depressive disorder

**DOI:** 10.1186/s12865-022-00479-3

**Published:** 2022-02-13

**Authors:** Linna Ning, Zhou Yang, Jie Chen, Zhaopeng Hu, Wenrui Jiang, Lixia Guo, Yan Xu, Huiming Li, Fanghua Xu, Dandong Deng

**Affiliations:** Department of Pathology, Gannan Medical University Pingxiang Hospital, Pingxiang, China

**Keywords:** Major depressive disorder, Immune-related genes, Immune infiltrates, CD3D, Logistic regression

## Abstract

**Background:**

Immune response is prevalently related with major depressive disorder (MDD) pathophysiology. However, the study on the relationship between immune-related genes (IRGs) and immune infiltrates of MDD remains scarce.

**Methods:**

We extracted expression data of 148 MDD patients from 2 cohorts, and systematically characterized differentially expressed IRGs by using limma package in R software. Then, the LASSO and multivariate logistic regression analysis was used to identify the most powerful IRGs. Next, we analyzed the relationship between IRGs and immune infiltrates of MDD. Finally, GSE76826 was used to to verificate of IRGs as a diagnostic markers in MDD.

**Results:**

203 different IRGs s in MDD has been identified (*P* < 0.05). GSEA revealed that the different IRGs was more likely to be enriched in immune-specific pathways. Then, a 9 IRGs was successfully established to predict MDD based on LASSO. Next, 4 IRGs was obtained by multivariate logistic regression analysis, and AUC for CD1C, SPP1, CD3D, CAMKK2, and IRGs model was 0.733, 0.767, 0.816, 0.800, and 0.861, suggesting that they have a good diagnostic performance. Furthermore, the proportion of T cells CD8, T cells γδ, macrophages M0, and NK cells resting in MDD group was lower than that in the healthy controls, suggesting that the immune system in MDD group is impaired. Simultaneously, CD3D was validated a reliable marker in MDD, and was positively correlated with T cells CD8. GSEA revealed high expression CD3D was more likely to be enriched in immune-specific pathways, and low expression CD3D was more likely to be enriched in glucose metabolism metabolism-specific pathways.

**Conclusions:**

We applied bioinformatics approaches to suggest that a 4 IRGs could serve as diagnostic markers to provide a novel direction to explore the pathogenesis of MDD.

**Supplementary Information:**

The online version contains supplementary material available at 10.1186/s12865-022-00479-3.

## Introduction

Major Depression Disorder (MDD) is an affective mental disorder syndrome with low mood, lack of interest and loss of fun as the main symptoms, accompanied by anxiety, cognitive impairment, psycho-motor disorder, and even suicide tendency [1–3]. The pathogenesis of MDD is complex, and is generally believed to be related to genetics, gender, neuroendocrine, psychosocial environment, immunity, intestinal microbes and other factors [4, 5]. Due to its high incidence, suicide rate, and disease burden, it has attracted wide attention from the government and researchers. At present, clinical diagnosis is mainly based on symptom description, mental state examination and clinical behavior observation of patients, lacking objective diagnostic indicators, which increases the misdiagnosis rate to a large extent [6]. Since modern drugs with selective serotonin reuptake inhibitor (SSRI), based on the monoamine neurotransmitter hypothesis, have shown only 50–60% efficacy in a large number of clinical studies [7, 8]. All of these problems hinder the diagnosis and treatment of MDD. As a result, there is an urgent need to develop more precise and individualized diagnosis and treatment methods.

Neuroimmunity as another important hypothesis in the pathogenesis of MDD has been paid attention to by researchers in recent years [9]. It has been confirmed that the brain has its own highly complex immune regulation system and is closely connected with the peripheral immune system [10]. Two-way communication between the immune system and CNS is crucial for establishing appropriate immunity to infection and injury, maintaining psycho-psychological and influencing behavioral responses [11, 12]. Despite extensive research on immune-related genes (IRGs), clinical research on the relationship between IRGs and MDD development is inadequate. The investigation of IRGs may provide new insights into the mechanisms of MDD development.

In this study, we integrated the transcriptome data of 148 MDD patients to comprehensively evaluate the biological patterns derived from IRGs, and systematically characterized differentially expressed IRGs by using limma package in R software. Then, the LASSO and multivariate logistic regression analysis was used to identify the most powerful IRGs. Next, we analyzed the relationship between IRGs and immune infiltrates of MDD. Finally, GSE76826 was used to to verificate of IRGs as a diagnostic markers in MDD.

## Materials and methods

### Data source and preprocessing

**Fig. 1:**
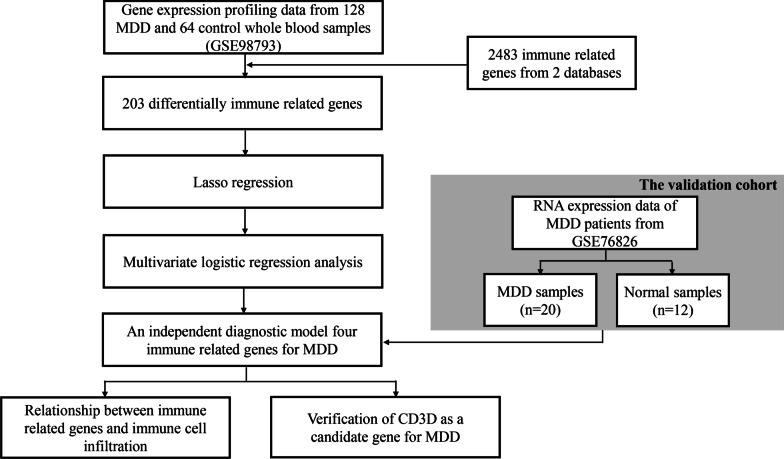
Flow chart of the steps in the performed analyses

The workflow of this study is shown in Fig. 1. We systematically searched MDD-related array datasets from the public databases and selected the MDD microarray data since 2010. the MDD microarray datasets were recruited from Gene Expression Ominibus (GEO) database (https://www.ncbi.nlm.nih.gov/geo/) with the following criteria: (1) only from Affymetrix platform; (2) diagnosed as MDD; (3) untreated patients; (4) the number of patients ≥ 10; (5) with more than 12,000 protein coding genes. Finally, GSE98793 (MDD: 128, 64 with generalised anxiety disorder, diagnosed by the MINI questionnaire, and 64 without anxiety disorder; healthy controls: 64) [13] and GSE76826 (MDD: 20; elderly (age ≥ 50 years) outpatients and inpatients with MDD corresponding to a DSM-IV diagnosis of the melancholy type of MDD episodes were studied. The depressive state was measured using the Structured Interview Guide for the Hamilton Depression (SIGH-D) rating scale. healthy controls: 12) [14] microarrays dataset were retrieved. We processed the raw data of these datasets using Robust Multi-array Average (RMA) method implemented in affy package for background adjustment, quantile normalization and final summarization of oligonucleotides per transcript via median polish algorithm [15]. Information on the data obtained is summarized in Table 1. Additionally, corresponding clinical information was extracted and manually organized either by directly downloading from the corresponding websites in GEO or by searching the published primary reports.

**Table 1:** Demographic characteristics of patients and healthy controls

	MDD (n = 128)	Control (n = 64)	χ^2^ or Z	*P* value
Age (years)	52.04 ± 11.51	52.03 ± 11.41	− 0.019	0.985
Gender			< 0.001	1.000
Male	32	16		
Female	96	48		

### Selection of different IRGs in MDD

A total of 1039 IRGs (removing the name-repeated gene) were downloaded from the InnateDB database (https://www.innatedb.com/) [16], and 2398 IRGs (removing the name-repeated gene) were downloaded from the ImmPort database (https://www.immport.org/shared/) [17]. Finally, a total of 1879 IRGs were finally identified (Additional file 1: Table S1). Use the “Limma” software package in the R statistical software to extract and analyze the downloaded data, and screen out the differentially expressed IRGs between MDD and normal [18]. We set the adjusted *P*-value < 0.05 as a significance threshold.

### Gene set enrichment analysis (GSEA)

We performed GSEA to identify differences in the enrichment of pathways and biological processes between different IRGs between MDD and normal. GSEA was conducted using the “clusterProfiler”, “enrichplot”, “patchwork”, and “DOSE” packages in R. We downloaded the gene sets of “c2.cp.kegg.v7.3.symbols” and “h.all.v7.3.symbols” from the MSigDB database for GSEA (http://www.gsea-msigdb.org/gsea/downloads.jsp). A significance level of 0.05 (FDR) was considered to indicate statistical significance [19].

### Construction of the diagnostic risk model

First, 203 different IRGs s in MDD has been identified (Additional file 2: Table S2). Then, to identify which genes are related to MDD, we analyzed the different IRGs by a Lasso regression model. This analysis revealed 9 significant genes. All the 9 genes were selected as feature genes to construct multivariate logistic regression analysis. Multivariate logistic regression analysis is one of the most widely used data dimensionality reduction algorithms that preserve the dimensional features with most of the differences. After obtaining the principal component coefficient of each sample, we applied a method similar to gene–gene interaction analysis to define the diagnostic score of each sample: (− 4.841 * expression level of CD1C) + (− 0.885 * expression level of SPP1) + (− 0.181 * expression level of CD3D) + (0.235 * expression level of CAMKK2).

### Immune cell infiltration

We performed the CIBERSORT method to calculate the enrichment scores on the basis of metagenes. CIBERSORT is a deconvolution algorithm that took a set of reference gene expression values as a minimum representation of each cell type, and based on these values, used support vector regression (SVR) to estimate the proportion of 22 immune cell types. The other was the Microenvironment Cell Populations-counter (MCP-counter) method, which estimated for population of 8 immune cell by contemplating the variations of the expression degree of one gene in a specific cell type and retained the genes showing the lowest variation within the cell type [20] (Additional file 3: Table S3).

### Correlation analysis between diagnostic markers and infiltrating immune cells

Spearman correlation analysis was performed on diagnostic markers and infiltrating immune cells, and the “ggplot2” package was used to visualize the results.

### Statistical analysis

The normality of the variables was evaluated using the Shapiro–Wilk normality test.Continuous variables between two groups were compared using the unpaired Student t-test and Mann–Whitney U test for parametric data and non-parametric data, respectively For comparison between more than two groups, we used parametric one-way ANOVA or non-parametric Kruskal–Wallis test. The receiver operating characteristic (ROC) curve was generated using the “pROC” package. All statistical analyses were two-sided and considered *P* < 0.05 as the threshold for statistical significance. The statistical results were all analyzed by R (version3.6.2).

## Result

### Identification of 203 differentially expressed IRGs

**Fig. 2:**
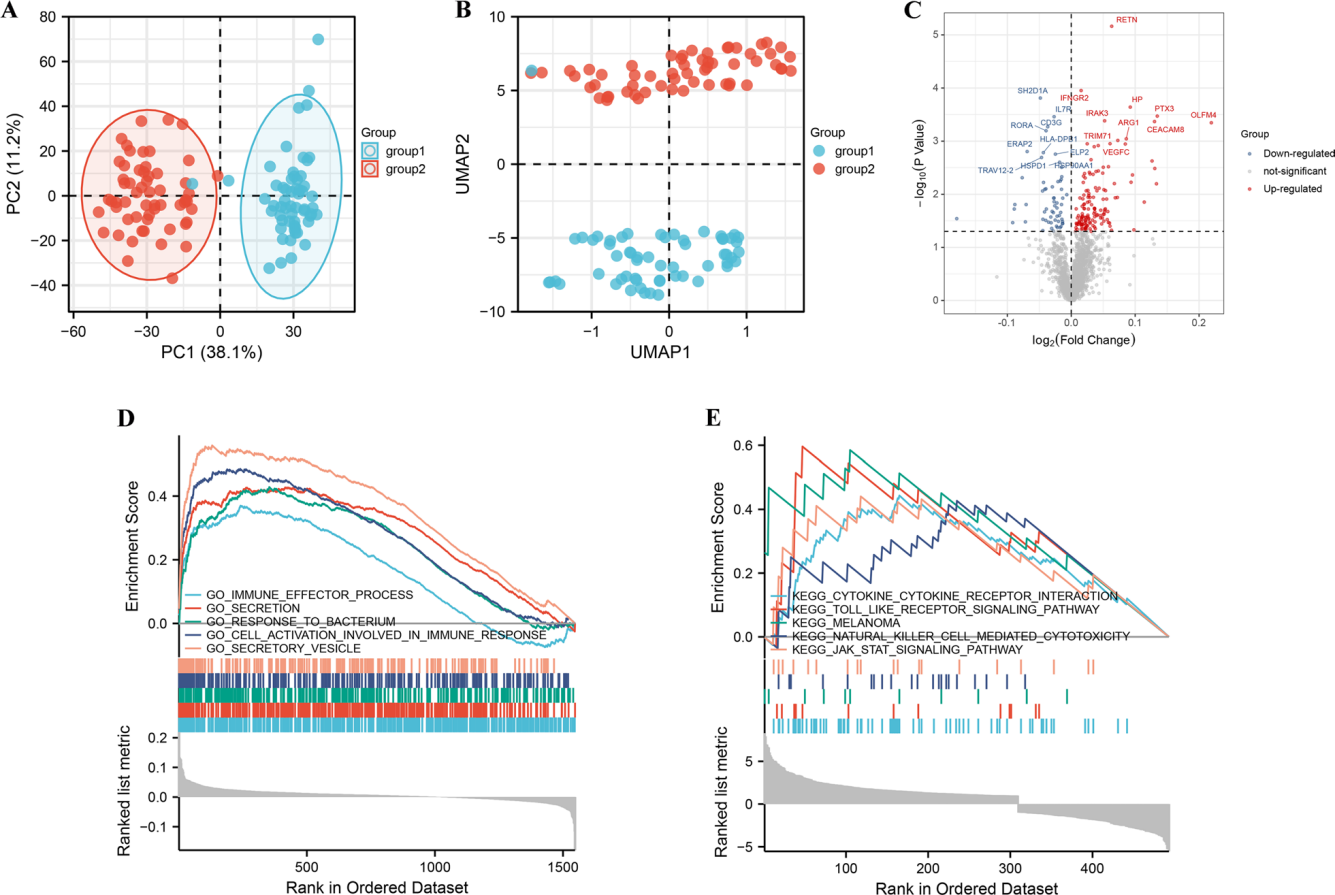
Identification of 203 differentially expressed IRGs. **A** Principal component analysis for the transcriptome profiles of MDD and healthy controls, showing a remarkable difference on transcriptome between different group. **B** UMAP analysis for the transcriptome profiles of MDD group and control group, showing a remarkable difference on transcriptome between different group. **C** Volcano plot of differentially expressed genes between MDD and healthy controls. Dot stands for gene. Red dots represent up-regulated genes, and green dots down-regulated genes. **D** GSEA GO identifies differentially expressed IRGs related signaling pathway in MDD. **E** GSEA KEGG identifies differentially expressed IRGs related signaling pathway in MDD

Base on ImmPort, InnateDB database, a total of 2483 IRGs were finally identified. Using 2483 IRGs, MDD group and healthy controls denoted a markedly discrimination each other, suggesting there are different IRGs between the two groups (Fig. 2A, B). According to the criteria that adj-*P*-value < 0.05, there are 203 differentially expressed IRGs, among which there are 142 up-regulated genes, and 61 down-regulated genes (Fig. 2C). Then, we performed GSEA to explore the biological process and pathway enrichment using the “hallmark” and “KEGG” gene sets for differentially expressed IRGs [21]. Hallmark gene sets comprehensively summarized specific well-defined biological processes and displayed high consistency in most published studies. As shown in Fig. 2D, E, differentially expressed IRGs were significantly enriched in immune-specific pathways. These data indicated that the pathogenesis of MDD may be involved in aforesaid biological processes.

### Construction of the diagnostic risk model

**Fig. 3:**
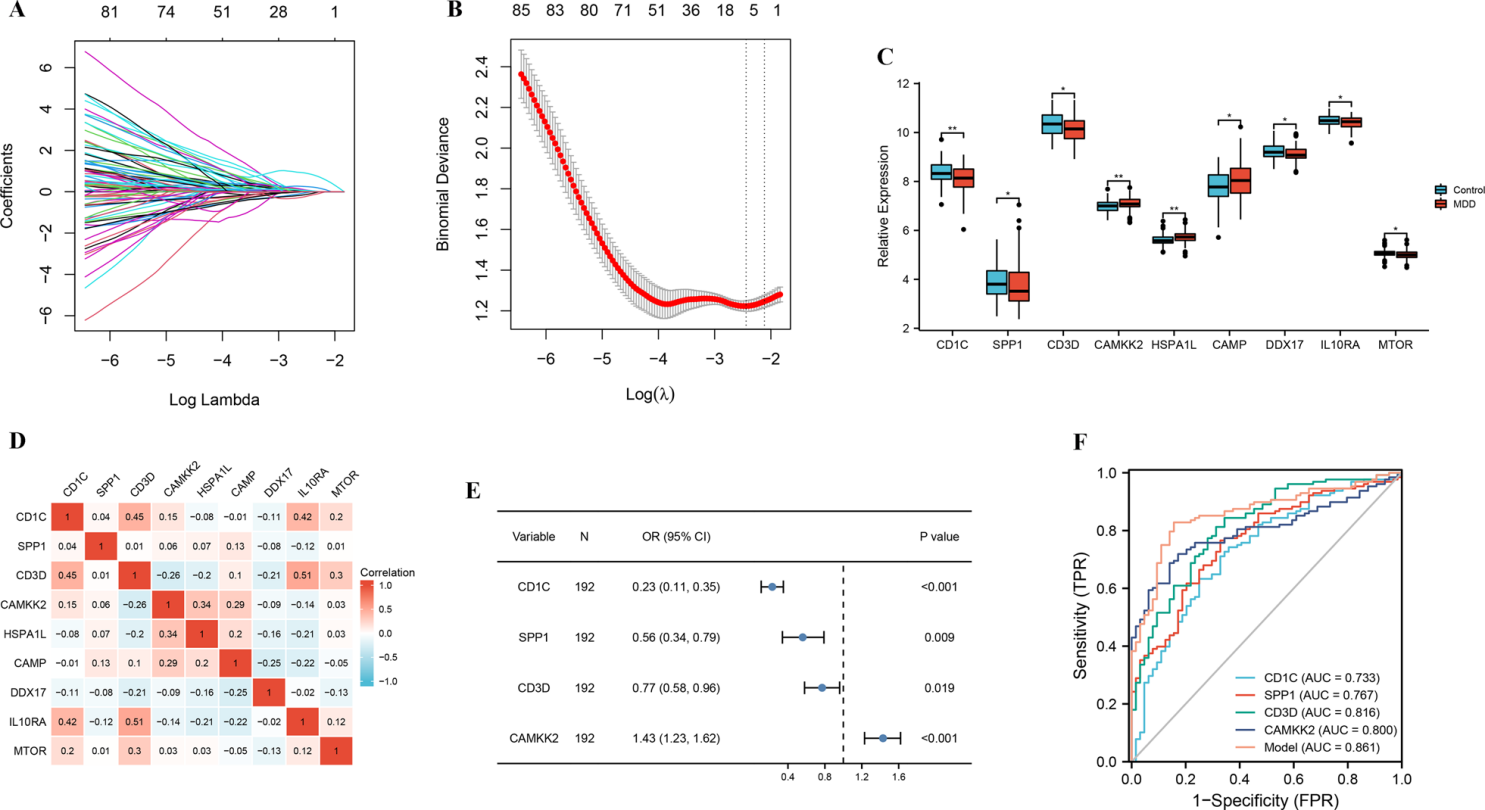
Construction of the diagnostic risk model. **A** Screening of the optimal parameter (using lambda.1se as the best lambda) at which the vertical lines were drawn. **B** LASSO coefficient profiles of the 9 differentially expressed IRGs. **C** Boxplots of 9 IRGs expression level in the MDD and healthy controls. **D** Correlation heat map of 9 differentially expressed IRGs. The size of the colored squares represents the strength of the correlation; blue represents a negative correlation, and red represents a positive correlation. The darker the color is, the stronger correlation is. **E** Multivariate logistic regression determined independent candidate diagnostic biomarkers. **F** ROC analysis showing that this diagnostic model has good diagnostic performance. Statistical analysis was performed using the Mann–Whitney U test

In order to identify the most powerful IRGs, we applied LASSO regression algorithm to the 203 differentially expressed IRGs. 9 IRGs were obtained (Fig. 3A, B). Moreover, the significant overexpressions of CAMKK2, HSPA1L, and CAMP in GSE98793 dataset were observed relative to normal control samples, while CD1C, SPP1, CD3D, DDX17, IL10RA, and MTOR were significantly lower in MDD group (Fig. 3C). Meanwhile, correlations of the 9 IRGs were also analyzed. CD3D was most correlated with IL10RA (r = 0.51) among all the interactions of 9 IRGs (Fig. 3D).

Next, all the 9 genes were selected as feature genes to construct multivariate logistic regression analysis (age and sex), and 4 IRGs (CD1C, SPP1, CD3D, and CAMKK2) was determined the independent candidate diagnostic biomarkers for MDD (Table 2). Of these, 3 IRGs were protective genes with odds ratio (OR) less than 1, except for the risky NSUN7 with OR larger than 1. Next, CD1C (OR = 0.23, 95%CI = 0.11–0.35), SPP1 (OR = 0.56, 95%CI = 0.34–0.79), CD3D (OR = 0.77, 95%CI = 0.58–0.96), and CAMKK2 (OR = 1.43, 95%CI = 1.23–1.62) determined the independent candidate diagnostic biomarkers for MDD (Fig. 3E). Moreover, the AUC of 0.733 for CD1C, 0.767 for SPP1, 0.816 for CD3D, and 0.800 for CAMKK2, suggesting that they have a good diagnostic performance. Furthermore, to improve the diagnostic efficiency of biomarkers, a novel diagnostic risk score was constructed by multiplying the gene expression of each gene and its corresponding coefficient, which was obtained by multivariate logistic regression analysis. The diagnostic ability of the IRGs model discriminating MDD from controls demonstrated a favorable diagnostic value, with an AUC of 0.861(Fig. 3F).

**Table 2:** Multivariate logistic regression analysis of immune related genes associated with major depressive disorder

Ensembl ID	Gene Symbol	Genomic location	Coefficient	OR	95% CI	*P* value
ENSG00000158481	CD1C	Chromosome 1: 158,289,923–158,294,774	− 4.841	0.23	0.11–0.35	< 0.001
ENSG00000118785	SPP1	Chromosome 4: 87,975,667–87,983,532	− 0.885	0.56	0.34–0.79	0.009
ENSG00000167286	CD3D	Chromosome 11: 118,339,075–118,342,705	− 0.181	0.77	0.58–0.96	0.019
ENSG00000110931	CAMKK2	Chromosome 12: 121,237,675–121,298,308	0.235	1.43	1.23–1.62	< 0.001

### Validation the 4 IRGs in GSE76826

**Fig. 4:**
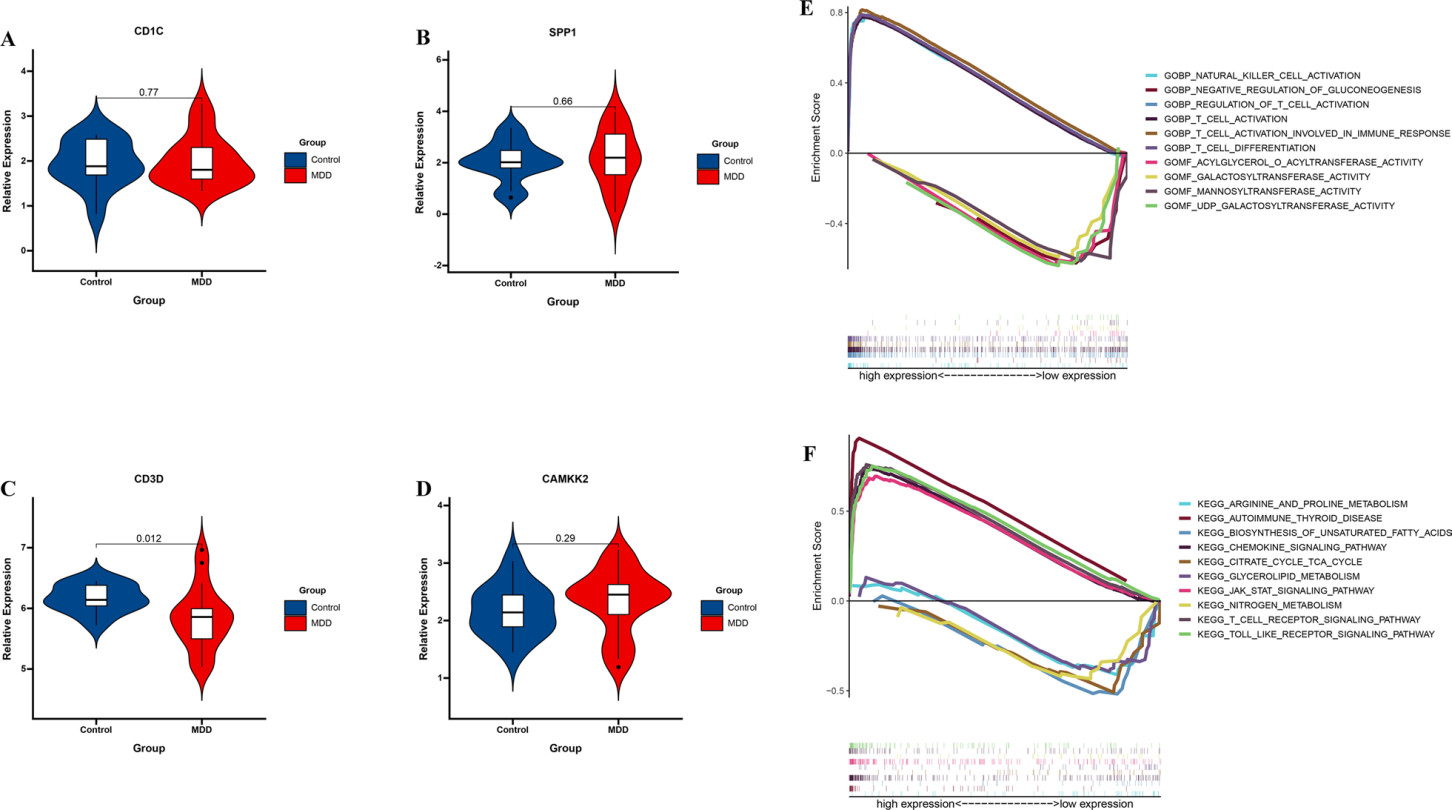
CD3D may represent a new candidate gene in MDD. **A** CD1C, **B** SPP1, **C** CD3D, and **D** CAMKK2 expression levels are shown for the MDD group and control group. **E** GSEA GO identifies high and low CD3D expression related signaling pathway in MDD. **F** GSEA KEGG identifies high and low CD3D expression related signaling pathway in MDD. Horizontal lines: median values. Statistical analysis was performed using the Mann–Whitney U test

To further validate 4 IRGs expression in MDD, GSE76826 cohort was used to measure the expression level of 4 IRGs expression, and the result showed that compared with normal group, the CD3D level was significantly lower in MDD group. However, there was no significant difference in CD1C (*P* = 0.77), SPP1 (*P* = 0.66), and CAMKK2 (*P* = 0.29) (Fig. 4A–D). These results support that CD3D was validated a reliable marker in MDD. Then, we performed GSEA to explore the biological process and pathway enrichment using the “hallmark” and “KEGG” gene sets for high and low CD3D expression. GSEA go revealed that the high CD3D expression group was enriched in T cell immune-related pathways, including T cell activation, regulation of T cell activation, T cell activation involved in inmune response, T cell differentition, and low CD3D expression group was enriched in glucose metabolism-related pathways, including negative regulation of gluconneogensis, galactosyltransferase activity, mannosyltransferrase activity, and UDP galactosyltransferase activity (Fig. 4E). GSEA KEGG revealed that the high CD3D expression group was enriched in TOLL like receptor signaling pathway, JAK STAT signaling pathway, T cell receptor signaling pathway, autoimune thyroid disease, chemokine signaling pathway, and low CD3D expression group was enriched in metabolism-related pathways, including biosynthsis of unsaturated fatty acids, steroid biosynthsis, arginine and proline metabolism, nitrogen metabolism, glycerolipid metabolism (Fig. 4F).

### Association of IRGs with immune infiltrates

**Fig. 5:**
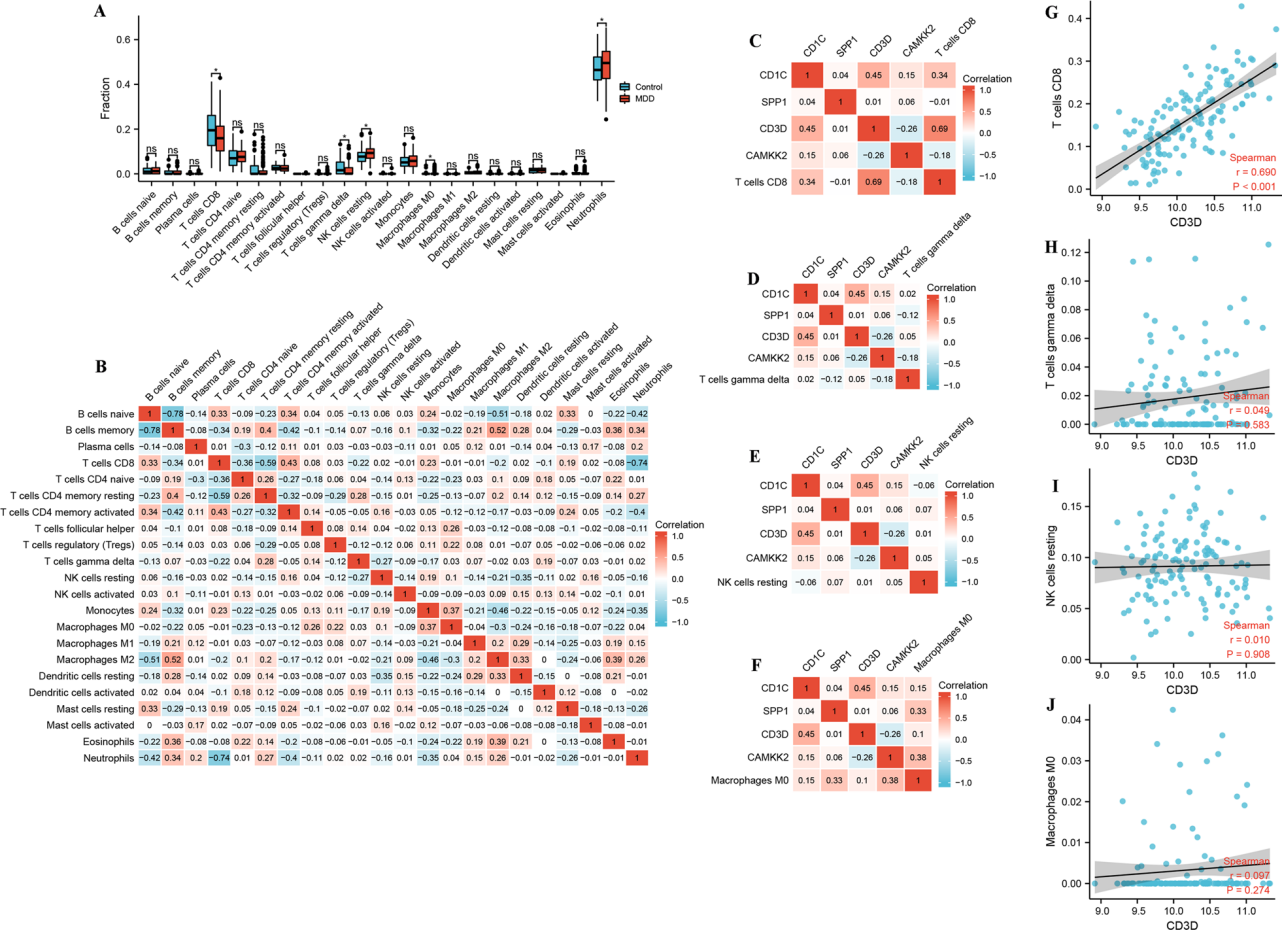
Association of IRGs with immune infiltrates. **A** The boxplots exhibits the differences in CIBERSOFT immune cell fractions between MDD and healthy controls. **B** Correlation heat map of 22 immune cell. The size of the colored squares represents the strength of the correlation; blue represents a negative correlation, and red represents a positive correlation. The darker the color is, the stronger correlation is. **C**–**F** Correlation heat map of 4 IRGs and immune cell. The size of the colored squares represents the strength of the correlation; blue represents a negative correlation, and red represents a positive correlation. The darker the color is, the stronger correlation is. **G**–**J** Correlation between CD3D and infiltrating immune cells

To gain further insight into the effects of the IRGs on immune infiltrates, we evaluated the correlation between IRGs and immune infiltrates in GSE98793 cohort. Firstly, by detecting immune infiltrates between the MDD group and healthy controls, we found that the proportion of T cells CD8, T cells γδ, macrophages M0, and NK cells resting in MDD group was lower than that in the healthy controls, suggesting that the immune system in MDD group is impaired. Simultaneously, the proportion of neutrophils in MDD group was higher than that in the healthy controls, suggesting that MDD may have an inflammatory response (Fig. 5A). Meanwhile, correlations of the 22 immune cells were also analyzed. B cells naive was most negative correlated with B cells memory (r = − 0.78) among all the interactions of 22 immune cells. B cells memory was most positive correlated with macrophages M2 (r = 0.52) among all the interactions of 22 immune cells (Fig. 5B). To gain further insight into the effects of the 4 IRGs expression on immune infiltrates, we evaluated the correlation between 4 IRGs expression and immune infiltrates in GSE98793 cohort. The results showed CD3D was positive correlated with T cells CD8 (r = 0.690, *P* < 0.05) (Fig. 5C–J).

## Discussion

Mounting evidence demonstrated that IRGs is closely related to hepatocellular carcinoma [22], gastric cancer [23], and ovarian cancer [24]. However, there is still a lack of comprehensive analyses of IRGs in psychiatric disorders. Luo et al. has showed IRGs (CXCL1) may be a diagnostic marker in epilepsy [25]. Thus far, the mechanisms underpinning the role of IRGs in MDD remain mostly unknown. This study aimed to systematically evaluated candidate signature IRGs to indicate diagnostic outcomes and investigate the correlations of immune infiltrates of MDD, which may assist us in evaluating diagnostic marker for MDD patients. In this study, we selected 2483 IRGs from two databases, and 203 different IRGs s in MDD has been identified in GSE98793 dadaset. Then, a 9 IRGs was successfully established to predict MDD based on LASSO. 4 IRGs (CD1C, SPP1, CD3D, CAMKK2) was obtained. CD3D showed highest AUC of the four genes, and the AUC of the diagnostic signature, and was validated by GSE76826 dadaset. GSEA revealed high expression CD3D was more likely to be enriched in immune-specific pathways, and low expression CD3D was more likely to be enriched in glucose metabolism metabolism-specific pathways. Furthermore, the AUC of 0.861 for IRGs model, suggesting that IRGs model have a good diagnostic performance. In addition, the proportion of T cells CD8, T cells γδ, macrophages M0, and NK cells resting in MDD group was lower than that in the healthy controls, suggesting that the immune system in MDD group is impaired. These results indicated that 4 IRGs and IRGs model showed a good performance and association with immune infiltrates, which maybe guide the selection of immunotherapy strategies against MDD.

Previous studies also reported some diagnostic biomarker signature in MDD. He et al. found that base on 4 autophagy-related signature have a good diagnostic performance in MDD (AUC = 0.779) [26]. By using machine learning methods, Zhao et al. constructed the classifiers of SVM, RF, kNN, and NB, and AUC for SVM, RF, kNN, and NB was 0.84, 0.81, 0.73, and 0.83, suggesting that they have a good diagnostic performance [27]. In this study, we found the AUC for IRGs model was 0.861, suggesting that IRGs model is better than the prediction performan of two recently published model.

Clinical depression is often associated with the change of immune response, including the number of circulating white blood cells, lymphocytes, neutrophil phagocytosis decreased, the phagocytosis of mononuclear cells increased, this a series of immune cells in the process of change, along with the numerous changes of immune inflammation factors, such as TNF-α, CRP, IL-6, SAA, and INF-γ [28, 29]. Changes in these inflammatory factors may be associated with disease, unhealthy lifestyle, or psychosocial stress [30]. Although their high expression predicts worsening of irritability symptoms in depression, it is not clear whether the effect of inflammation on depression increases over time [31]. Presumably, there is a bidirectional relationship between depressive symptoms and inflammation. In this study, the proportion of neutrophils in MDD group was higher than that in the healthy controls. This result confirms the relationship between depression and inflammation.

Mounting evidence of immune imbalance in MDD has sprung up in recent decades. For example, immune cell counts, especially neutrophils, CD4+ T cells and monocytes, are increased in the whole blood of MDD patients compared to healthy controls. However, the activation pattern of immune cells in MDD is still unclear. To further explore the role of immune cell infiltration in MDD, we used CIBERSORT to conduct a comprehensive evaluation of MDD immune infiltration. Our results found that the proportions of T cells CD8, T cells γδ, macrophages M0, and NK cells resting in MDD patients were significantly lower than those in controls. Immune infiltration of resting NK cells in MDD has not been previously reported.

T cells increased significantly in the brain of MDD mice [32]. These cells were concentrated in the gray matter region of MDD mice at 12 months of age, and widely distributed in the brain of MDD mice at 22–24 months of age [33]. CD8^+^ CD45RA^+^ T cell subsets are hyperactivated cells that can further damage neurodegenerative individuals by releasing inflammatory molecules or by direct contact with neurons themselves resulting in neuronal dysfunction [34]. Previous studies have shown that CD8+ T cells are negatively correlated with cognition. CD8^+^ T cells in the peripheral immune system were significantly reduced in MDD patients [35]. Our result also confirms this relationship.

The immune system is regulated by both costimulatory signaling molecules and inhibitory molecules, known as immune checkpoints. T-cell activation requires two signals, The first signal is antigen presenting cell (APC) through antigen peptide/major histocompatibility complex (MHC) and T cell receptor, T cells and APC costimulatory signals serve as the second signal of T cell activation, stimulating checkpoints promote initial T cell activation as well as effector cell, memory and regulatory T cell (Treg) responses. Inhibitory checkpoint limits the threshold of T cell activation, shortens the duration of immune response, and plays a role in regulating inflammation and tolerance [36]. CD3 consists of different strands (γ, δ and ε) encoded by CD3G, CD3D and CD3E. These chains can bind to TCR and ζ chains (encoded by CD3Z) to form TCR-CD3 complexes, which play an important role in T cell antigen recognition and signal transduction. They all have their own unique functions in addition to the role of signal transduction in T cell activation [37]. CD3D had an impact on the phenotype and in vitro function of immune cells. Previous study has showed that CD3D is closely related to glioblastoma multiforme [38], cervical cancer [39], and breast cancer [40]. Another study showed that CD3D was the target of mir-182-5p and might be used as candidate biomarkers of breast cancer [41]. However, there is still a lack of comprehensive analyses of CD3D in MDD.

Our study has some limitations. First, given the individual heterogeneity of MDD, the results of our study should be further validated using more multicenter clinical data. Last, our findings have substantial implications for IRGs of MDD, and the detailed molecular mechanisms require further research to explore deeper interactions.

In a conclusion, this study was focused on the analysis of IRGs in MDD, and found 4 IRGs have a good diagnostic performance in MDD. Based on the 4 IRGs, we constructed the IRGs model, and the diagnostic ability of the IRGs model is reliable. In addition, the proportion of immune infiltrates in MDD group was lower than that in the healthy controls. Simultaneously, CD3D was positively correlated with immune infiltrates. These results indicated that 4 IRGs and IRGs model showed a good performance and association with immune infiltrates, which maybe guide the selection of immunotherapy strategies against MDD.

## Supplementary Information

**Additional file 1: Table S1.** The expression of 1879 IRGs in GSE98793 dataset.

**Additional file 2: Table S2.** Differentially expressed IRGs between MDD and healthy controls in GSE98793 dataset.

**Additional file 3: Table S3.** The proportion of 22 immune cells in GSE98793 dataset.

## Data Availability

The datasets in the current study come from Gene Expression Ominibus (GEO) database (https://www.ncbi.nlm.nih.gov/geo/): GSE98793 and GSE76826.
